# Psychological Capital and Job Satisfaction Among Chinese Residents: A Moderated Mediation of Organizational Identification and Income Level

**DOI:** 10.3389/fpsyg.2021.719230

**Published:** 2021-10-11

**Authors:** Fang Zhang, Ying Liu, Tongqi Wei

**Affiliations:** ^1^Beijing Children’s Hospital, Capital Medical University, Beijing, China; ^2^School of Medical Humanities, Capital Medical University, Beijing, China; ^3^Institute of Psychology, Chinese Academy of Sciences, Beijing, China

**Keywords:** organizational identification, psychological capital, job satisfaction, income level, residents

## Abstract

The present study examined the mediating effect of organizational identification on the relationship between psychological capital and job satisfaction, and whether the mediation was moderated by income level. A total of 310 Chinese residents were surveyed using the Psychological Capital Scale, Organizational Identification Scale, Job Satisfaction Scale, and a demographic questionnaire. The findings showed a significant positive correlation between psychological capital and job satisfaction of residents, and this relationship was partially mediated by organizational identification. Moreover, income level played a moderating role in the relationship between organizational identification and job satisfaction. For residents with more income, their organizational identification influenced their job satisfaction more strongly than those with less income. The current study contributes to a better understanding of the relationship between psychological capital and job satisfaction. Implications for resident management and policymaking are discussed.

## Introduction

Residents (also called resident physicians) are a special group of physicians who work in hospitals. They have received formal education in medical schools and must receive special clinical training at the hospital and under the supervision of the medical staff for 2–7 years before they can work independently. The job responsibilities for most of the residents in China involve taking care of hospitalized patients. They are one of the groups that have the closest contact with patients and are under enormous work stress (Chen et al., 2013). Compared with attending surgeons, residents were found to have a higher risk of burnout and poorer quality of life (Pulcrano et al., 2016). Moreover, the turnover intention of this group of employees is much higher. A systematic literature review concluded that the burnout of residents could be traced back to the period when they were medical students, and the main reasons contributing to burnout were a lack of time control and heavy workload (IsHak et al., 2009). In this situation, it is critical to pay more attention to the resident population, helping them avoid the negative effects of work stress and gain more happiness from the work they do.

As a group of employees with relatively less seniority, building identification with the organization for residents is essential to improve work attitudes and behaviors. Research has proven that organizational identification has a positive effect on job satisfaction, and organizational commitment behaviors. A variety of studies have established that organization and environment level factors, such as organizational climate, perceived support from the organization, and the autonomy of the job contribute to employees’ organizational identification (Edwards and Peccei, 2010; Salvatore et al., 2018; Barattucci et al., 2021). However, little attention has been paid to the intrinsic positive power of individuals that can impact on organizational identification.

In Chinese employees, Luthans et al. (2008a) identified psychological capital as a core psychological element of individual motivation and asserted that it is of critical importance in influencing performance, positive attitudes, and positive behaviors. Previous studies have shown that both identification and psychological capital are predictors of job satisfaction (Larson and Luthans, 2006; van Knippenberg and Sleebos, 2006; Ke and Sun, 2014; Loi et al., 2014; Karanika-Murray et al., 2015). However, the interaction between explaining and predicting such a mechanism is less clear. We propose that psychological capital is associated with job satisfaction and that this relationship is mediated by organizational identification. Meanwhile, we focus on income level as an important factor related to job satisfaction and explore whether it moderates the relationship between organizational identification and job satisfaction. We conducted this study to generate fresh insights into improving junior employees’ positive perceptions of their jobs. We believe that these findings make an important Contribution To The Field of positive organizational behavior.

### Theory and Hypothesis

#### The Positive Impact of Psychological Capital on Job Satisfaction

Job satisfaction is a work-related attitude that reflects how satisfied or dissatisfied an employee is with their job (Spector, 1985). Job satisfaction has been noted by many researchers as critical for hospital staff; for example, it has been shown to relate to many significant aspects of job performance, such as work engagement and burnout, turnover intention, emphatic behaviors toward patients, and patient satisfaction (Iyeke, 2020; Yu et al., 2020; Schneider et al., 2021). Furthermore, the effects of job satisfaction in medical personnel are reflected not only in the organizational aspects, but also in their mental and physical health conditions (Verbrugge, 1982; Cooper et al., 1989; Williams et al., 2001).

Among many individual and organizational factors that shed light on job satisfaction, one of the most important individual factors is psychological capital. Luthans et al. (2005) defined psychological capital (hereinafter PsyCap) as the core psychological element of individual motivation, including self-efficacy, optimism, resilience, and hope. Self-efficacy is defined as people’s confidence about their abilities to deploy the inspiration, cognitive resources, and action plan needed to successfully complete a specific task (Stajkovic and Luthans, 1998). It was derived primarily from Bandura (1997) social cognition theory, and it is about the perception that a person can take effective action to achieve a desired goal. Resilience is defined as “the capacity to rebound or bounce back from adversity, conflict, failure or even positive events, progress, and increased responsibility” (Luthans, 2002, p. 702). It is an intrinsic resource that enables individuals to protect themselves from the negative effects of the stressors they encounter by changing their responses to adversity (Fletcher and Sarkar, 2013). Optimism is a positive attribution style that attributes positive results to personal and pervasive elements, and interprets negative events with reference to external or environmental causes (Luthans and Youssef-Morgan, 2017). Hope is defined as “a positive motivational state based on an interactively derived sense of successful agency and pathways” (Snyder et al., 1991). It mainly focuses on one’s determination to pursue goals and the ability to find alternative solutions to achieve the goal. The four resources combine into a higher-order core construct named PsyCap, which satisfies the criteria of positive organizational behavior and enables individuals to gain a competitive advantage through targeted investment and development.

Studies have shown that PsyCap played an important protective role in the workplace when associated with work-related stress (Khalid et al., 2020; Tian et al., 2020). Moreover, people with higher levels of PsyCap were more positive and motivated (Luthans et al., 2008b), demonstrating more organizational citizenship behavior (Gupta et al., 2017) and voice behavior (Wang et al., 2018) in the workplace. Evidence also shows that the impact of PsyCap on employees’ work attitudes is more pronounced than that of human and social capital (Larson and Luthans, 2006). Due to the lack of clinical experience, residents need to gain a lot of practical knowledge in their work, and self-efficacy can help them master the required knowledge and skills quickly. In addition, optimistic residents can regulate their emotional states, which also helps them to do their jobs with more positivity. In the current study, it was predicted that residents with higher levels of PsyCap will be more satisfied with their jobs (H1).

#### The Mediating Effect of Residents’ Organizational Identification in the Relationship Between Psychological Capital and Job Satisfaction

Ashforth and Mael (1989) defined organizational identification (hereafter OID) as the extent to which an organization’s members define themselves by their membership in the organization. Rooted in social identity theory, OID applies this theory to an organizational context. Social identity is a part of an individual’s self-concept, which comes from the individual’s perception of being a member of a social group, and sharing certain values with the group and its members. As part of a particular social group, employees also identify with their groups or organizations accordingly, which represents their acceptance of the organization’s value and promises their organizational commitment in the future (Barattucci et al., 2021). Lee et al. (2015) concluded in their meta-analysis that OID exists as the basis for general attitudes and behaviors in organizations. When employees identify themselves with the organization, they tend to have a higher level of commitment, loyalty, and more positive attitudes toward the organization. Several studies describe the role of OID in the job satisfaction of employees from varied vocations (Van Dick et al., 2004; Loi et al., 2014; Lee et al., 2015; Lu et al., 2015). Altogether, we hypothesized that residents’ OID would positively predict their job satisfaction (H2).

Most studies that focus on these two concepts believe that they play a joint role in influencing employees’ job satisfaction. However, studies exploring the relationship between PsyCap and OID are rare, and the interaction between these two concepts remains unclear. From the limited literature, we obtained two contradictory claims. On one hand, some researchers believe that OID influences PsyCap (Lu et al., 2015). The employee establishes a relationship with the organization through an employment contract in the early stages of the job. As the work continues, employees build their identity with the organization. When employees recognize that their relationship with the organization is positive, it will enhance their sense of self-efficacy, optimism, and other positive mindsets (Chen et al., 2017). Nevertheless, this sort of explanation lacks theoretical support.

On the other hand, more research has concluded the opposite relationship, that PsyCap induces OID. First, the Conservation of Resources (COR) Theory suggests that individuals with more resources tend to be less susceptible to the loss of resources, and to be more capable of gaining more resources (Hobfoll, 2001). As a positive psychological resource, people with higher level of PsyCap can get protection against the threat caused by the decline of OID and they are more likely to find more identity with the organizations, for recognition of OID is a kind of cognition resource for the employees. Consequently, an increase in PsyCap will contribute to the improvement of employees’ OID (Yang and Chao, 2016).

Second, according to Fredrickson (2001) broaden-and-build theory of positive emotions, resilience can motivate employees to face the organization with a more positive mindset and put more effort into their work, thus making it easier for them to identify with the organization (Wu and Zhang, 2017).

Taking these two different views into account, this study argues that PsyCap affects OID, not the reverse. Our inference was mainly based on the explanations from the broaden-and-build theory and resource conservation theory mentioned above. Since both of the two factors have an impact on job satisfaction, we hypothesized in current research that OID would play a mediating role in the impact of residents’ PsyCap on their job satisfaction (H3).

#### The Moderating Effect of Income Level

It has become common knowledge that residents around the world are treated poorly. The Medscape Residents Salary & Debt Report for 2021 indicated that more than 1/3 of residents are dissatisfied with their salaries because they do not reflect their actual working hours and are not comparable to the salaries of other medical staff (Martin, 2021). Current research has yet to conclude whether a low level of income affects residents’ job satisfaction. In his dual-factor theory, Herzberg suggested that salary could not lead to better job satisfaction (Brenner et al., 1971). This assertion has been confirmed in some studies; for example, one study has indicated that income level is only weakly associated with workers’ satisfaction (Clark and Oswald, 1996). On the other hand, researchers have found that income is one of the most influential factors on the satisfaction of medical doctors’ jobs (Sharma and Bajpai, 2011; Vuong et al., 2021). The main reason for this controversy is that little research has taken into consideration the effect of OID and income level together. It is impossible for employees who only have a low level of OID to feel satisfied with their jobs, regardless of how much their salaries are. Income has something to do with job satisfaction only on the premise that the employee has identified with the organization. From this point of view, we hypothesize that income level might be a moderator in the relationship between OID and job satisfaction. The positive correlation between OID and job satisfaction increases as income level rises (H4).

#### The Present Study

**FIGURE 1:**
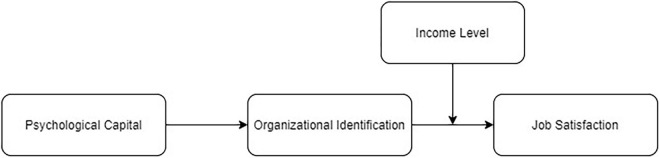
Hypothesized model.

This study focused on the relationship between residents’ PsyCap and job satisfaction, as well as the mediator role played by OID. We also examine the moderating effect of income level on the relationship between OID and job satisfaction. We hypothesize that OID plays a mediating role between PsyCap and job satisfaction, whereas income level moderates the relationship between OID and job satisfaction. Based on the theories and literature reviewed above, we propose a theoretical model in Figure 1 regarding the mechanism effect of residents’ PsyCap on their job satisfaction. Specifically, it is assumed that residents who develop a higher PsyCap identify more with their organization, which in turn will increase their level of job satisfaction. Meanwhile, the influence of OID will be greater for residents with a higher income level than for those with less income.

## Materials and Methods

### Participants

Participants were recruited during standardized training in a medical university of residents from public hospitals. Sampling took place twice, 1 year apart. The residents who attended the training did not attend it again, ensuring that there were no duplicates in the sample. Recruitment procedures were identical in order to ensure the standardization of the sampling process. A verbal explanation of the purpose and the anonymity of the project was given at the break time between training classes. Residents who were willing to participate in the research gave their informed consent and were given the questionnaires after the class. In two samplings, 340 residents participated altogether. We obtained 153 participants through the first sample, and 157 in the second. These two samples were combined into one sample because their demographics were not significantly different (*p* > 0.05). Thirty participants were excluded from the analyses due to the high repetition rates of their responses, which reflected their lack of seriousness in answering the questionnaires. The remaining 310 participants consisted of 223 women (72.9%), 84 men (28.1%), and three participants (0.01%) who did not identify their gender.

### Measurement

The materials consisted of a battery of tests covering organizational identification, psychological capital, job satisfaction, and demographic items. Each scale is described in detail below.

#### Psychological Capital

Psychological capital was measured using the Chinese version of the Psychological Capital Questionnaire (PCQ) translated by Chaoping Li (Luthans et al., 2008c). The validity of the Chinese version of the PCQ has been examined by several researchers. This questionnaire was also used by the originators of the psychological capital theory in a mainland Chinese sample (Luthans et al., 2005). The questionnaire contained four dimensions: self-efficacy, optimism, hope, and resilience, with six items in each subscale. A six-point Likert scale was adopted to identify residents’ PsyCap, with higher total scores representing a higher level of PsyCap, and the average score for each item was calculated. Luthans et al. (2007) emphasized that PsyCap is a higher-order structure that should be viewed and studied as a whole. Therefore, this study considers psychological capital as a whole construct. In the current study, the consistency coefficient of the PsyCap questionnaire was 0.90.

#### Organizational Identification

We used a revised version of the Organizational Identification Scale developed by Mael and Ashforth (1988) and Mael and Tetrick (1992) to measure participants’ identification with their hospitals. This scale is unidimensional and has high structural and internal consistency reliability. We replaced the phrase “name of school” with “my hospital.” An example item is “When someone praises my hospital, it feels like a personal compliment.” The questionnaire consisted of six items. Each item was rated on a 5-point scale ranging from 1 (strongly disagree) to 5 (strongly agree). The higher the total score, the stronger the individuals identified with their organization. This test showed an internal consistency of 0.81 (Cronbach’s alpha).

Job satisfaction is measured by a single item that asked participants, “In general, how satisfied are you with the current job?” with a 5-point scale where 1 means not at all unsatisfied, and 5 means extremely satisfied. The single-item measure of job satisfaction has been confirmed to be comparable to the multiple-items measure of job satisfaction (Wanous et al., 1997; Dolbier et al., 2005).

#### Demographic Items

The demographic questionnaire included age, gender, education level, years worked, and income per month.

## Results

First, we performed confirmatory factor analysis using AMOS 23.0 to examine the discriminant validity of the core variables and to test the common method variance. Then, we used SPSS 23.0 to statistically analyze the means, standard deviations, correlations, and reliability of the measures for each variable. Finally, we used SPSS 23.0 to perform path analysis to test the theoretical model as a whole.

### Confirmatory Factor Analysis

For the structural validity of the four variables of PsyCap, OID, job satisfaction, and income level, this study conducted a confirmatory factor analysis using Amos 23.0, and compared the model fit indices with other models. The results of the analysis are shown in Table 1. It indicates that the four-factor model fits best with the existing data, and each fitting index meets the criteria, which proves the uniqueness of the four variables.

**TABLE 1:** Results of confirmatory factor analysis.

Models	χ^2^	df	TLI	CFI	RMSEA	SRMR	Model comparison		
								Δχ^2^	Δdf
1. Baseline model	117.091	47	0.915	0.939	0.069	0.064			
2. Three-factor model 1	282.413	50	0.734	0.798	0.123	0.106	2 vs. 1	165.322***	3
3. Three-factor model 2	562.892	49	0.815	0.399	0.554	0.155	3 vs. 1	445.801***	2
4. Two-factor model	277.415	48	0.726	0.801	0.124	0.103	4 vs. 1	160.423***	1
5. Single-factor model	323.184	50	0.687	0.763	0.133	0.114	5 vs. 1	206.093***	3

*Baseline (four-factor model): Psycap, OID, JS, Income; Three-factor model 1: Psycap + OID, JS, Income; Three-factor model 2: Psycap + JS, OID, Income; Two-factor model: Psycap + OID, JS + Income; Single-factor model: Psycap + OID + JS + Income.*

N = 310, Δχ^2^ is the change of χ^2^ compared with the baseline model. PsyCap, Psychological Capital; OID, Organizational Identification; JS, Job Satisfaction. ***P < 0.001.

### Common Method Bias Test

First, the data of the present study were collected at two different times, which helped to decrease the possibility of common method bias (CMB). Second, two statistical methods were used to test CMB. Harman’s single-factor test (Podsakoff et al., 2003) was performed, and the results showed that the KMO value was 0.88 (*p* < 0.001), indicating that the scales were suitable for factor analysis. There were seven factors with eigenvalues >1, and the first factor explained a variance of 29.98%, which did Not reach the critical criterion of 40%. Moreover, the result of single latent factor confirmatory factor analysis (Table 1) showed that the fitting indices of the one-factor structure model were poor (χ^2^/*df* = 6.46, CFI = 0.76, TLI = 0.69, RMSEA = 0.13), which indicates no serious CMB issues in this study.

### Demographic Characteristics

The average age of participants was 27.03 years (*SD* = 2.63), and 84.19% had a bachelor’s degree or higher education. The average years worked was 1.71 years (*SD* = 1.36). Furthermore, 3.4% of the residents earned a monthly income exceeding 10,000 RMB, while 77.4% earned less than 6,000 RMB each month.

### Descriptive Statistics and Correlation Analysis

The means, SDs, Cronbach’s alpha, composite reliability (CR), square roots of the average variance extracted (AVE), and correlation coefficients are presented in Table 2. The internal consistency coefficients ranging from 0.71 to 0.91 indicated acceptable reliability, CR exceeded the threshold of 0.7 and AVE exceeded 0.5, which promise a good convergent validity (Fornell and Larcker, 1981; Segars, 1997). Analysis revealed that PsyCap was positively related to job satisfaction (*r* = 0.41, *p* < 0.01) and OID (*r* = 0.30, *p* < 0.01). OID was positively related to job satisfaction (*r* = 0.34, *p* < 0.01), supporting Hypotheses 1 and 2. However, there was no significant correlation between income and the other three variables (*r* = -0.03 to 0.08, *p* > 0.05). These results provide preliminary evidence for the Hypotheses 3 and 4.

**TABLE 2:** Descriptive statistics and correlations between study variables.

	*M*	*SD*	*Cronbach’s* α	*CR*	*1*	*2*	*3*
Psycap	4.22	0.64	0.91	0.85	**0.77**		
OID	3.63	0.65	0.71	0.71	0.30**	**0.58**	
Income	1.89	1.03	–	–	0.02	–0.03	–
Job satisfaction	3.33	0.92	–	–	0.34**	0.41**	0.08

N = 310, **p < 0.01. Square-roots of the AVEs appear in bold along the diagonal of the correlation of constructs.

### The Moderated Mediating Effects

We adopted the suggestion of Edwards and Lambert (2007) and did an analysis using the bootstrap method. Using the PROCESS Macros Model 4 and 14 developed by Hayes (2013), we examined the moderated mediating effect of OID on the relationship between PsyCap and job satisfaction, while controlling for the participants’ gender, age, education level, and the years worked.

The results (see Table 3) showed that PsyCap had a significant positive effect on job satisfaction in the absence of mediating variables (β = 0.40, *t* = 7.61, *p* < 0.001), supporting Hypothesis 1. PsyCap also had a significant positive effect on OID (β = 0.29, *t* = 5.13, *p* < 0.001), supporting Hypothesis 2. After adding the mediating variable of OID, the positive effect of PsyCap on job satisfaction remained significant (β = 0.36, *t* = 6.26, *p* < 0.001), and the results of the mediation effect test using Bootstrap showed that, in terms of the direct effect, the 95% confidence interval for Psycap on job satisfaction ranged from 0.22 to 0.44. The mediating effect of OID was 0.07 (0.29 × 0.25), and the 95% confidence interval did not contain zero (from 0.03 to 0.12), which indicates that the mediation effect was satisfied, accounting for 17.5% of the total effect (0.07/0.40). The establishment of the mediation effect suggests that PsyCap not only directly affects residents’ job satisfaction, but also indirectly affects their job satisfaction through OID. Thus, Hypothesis 3 was supported.

**TABLE 3:** Hierarchical regression results.

Dependent variable	Model 1 (Job satisfaction)			Model 2 (OID)			Model 3 (Job satisfaction)			Model 4 (Job satisfaction)		
				
	β	t	Boot 95% CI	β	t	Boot 95% CI	β	t	Boot 95% CI	β	t	Boot 95% CI
Gender	–0.05	–1.00	[−0.37, 0.12]	0.02	0.33	[−0.21, 0.29]	–0.06	–1.06	[−0.36, 0.09]	–0.12	–1.05	[−0.36, 0.09]
Age	0.11	1.64	[−0.01, 0.08]	0.07	1.06	[−0.02, 0.08]	0.09	1.50	[−0.01, 0.08]	0.04	1.50	[−0.01, 0.08]
Edu level	0.02	0.42	[−0.10, 0.17]	–0.08	–1.36	[−0.24, 0.04]	0.05	0.06	[−0.07, 0.19]	0.00	0.06	[−0.14, 0.15]
Years worked	–0.05	–0.78	[−0.14, 0.08]	–0.10	–1.69	[−0.18, 0.01]	–0.02	–0.69	[−0.11, 0.07]	–0.03	–0.69	[−0.12, 0.06]
Psycap	0.40***	7.61	[0.28, 0.52]	0.29***	5.13	[0.18, 0.40]	0.36***	6.26	[0.22, 0.44]	0.33***	6.10	[0.22, 0.43]
OID							0.25***	4.66	[0.14, 0.36]	0.25***	4.70	[0.14, 0.35]
Income										0.10	1.84	[−0.01, 0.21]
OID*Income										0.10*	2.14	[0.01, 0.19]
*R2*	0.18			0.10			0.23			0.25		
*F*	12.60***			6.47***			15.02***			12.46***		

*CI, confidence interval, it represents significance when the confidence intervals did not contain zero.*

*p < 0.05; ***p < 0.001.

**FIGURE 2:**
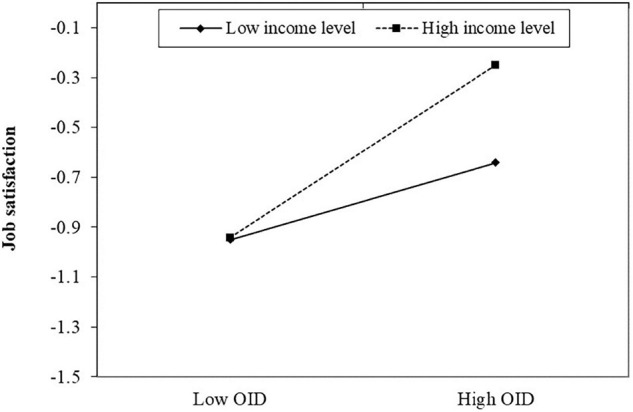
Simple slopes of the moderation.

Moreover, the product of income level and OID had additional effect on job satisfaction (β = 0.10, *p* < 0.05) (Table 3). Simple slope test (Figure 2) showed that the increase in income level enhanced the positive effect of OID on job satisfaction. As seen in Table 4, there is a difference in the indirect effect of OID at the high and low levels of income (ΔB = 0.06). The mediating effect of PsyCap on job satisfaction through OID was significant at high income levels (M + SD) (β = 0.10, *p* < 0.05) with 95% CI of [0.04, 0.16], while the mediating effect of OID was not significant (β = 0.04, *p* > 0.05) at low-income levels (M-SD), with 95% CI including 0. Hypothesis 4 was supported.

**TABLE 4:** Conditional indirect effect of income level when organizational identification (OID) mediated between PsyCap and job satisfaction.

Mediator	Income level	Effect	BootSE	95%CI
OID	–1 SD	0.04	0.03	[−0.00, 0.01]
	M	0.07	0.02	[0.03, 0.12]
	+1 SD	0.10	0.03	[0.04, 0.16]

## Discussion

In this study, we explored the relationship between PsyCap, OID, and job satisfaction, and probed the underlying mediation and moderation mechanisms. We established a moderated mediation model to test whether residents’ PsyCap was indirectly associated with job satisfaction through OID and whether this indirect relationship was moderated by income level. The results indicated that the impact of PsyCap on residents’ job satisfaction can be partially explained by their identification with the organization. Furthermore, this indirect relationship was moderated by the income level in the second stage of the mediation process, such that the effect of OID on job satisfaction was stronger in the context of a higher level of income. In other words, the positive relationship between OID and job satisfaction strengthens as income level increases.

The positive relationships between PsyCap and job satisfaction, and OID and job satisfaction have been proven in several studies (Larson and Luthans, 2006; Ke and Sun, 2014; Loi et al., 2014; Karanika-Murray et al., 2015). The duplicated result reminds us of the importance of an employee’s psychological resources, and their identification with the organizations they are working for.

As mentioned in the literature review, OID has a significant influence on job satisfaction. However, little is known about how to improve the OID of employees (especially in newcomers). The results of the current study shed new light on this issue by introducing PsyCap as a positive psychological resource. We argue that PsyCap not only has a direct effect on job satisfaction, but it also influences job satisfaction through the mediating role of OID. It is well-established that an individual’s attitude toward life and work is profoundly affected by PsyCap. Employees with less PsyCap are more likely to experience helplessness, anxiety, depression, and frustration, and thus have more negative experiences at work. It may also cause them to disagree with and distrust the organization, which leads to distress. On the other hand, people with higher levels of PsyCap are more positive. The broaden-and-build theory suggests that positive emotions have two main functions: broadening and building. The broadening function refers to the ability of positive emotions to expand the scope of attention, cognition, and action. Building function refers to the ability of positive emotions to build enduring resources for individuals (Fredrickson, 2001). Therefore, positive emotions resulting from high levels of PsyCap would help residents to be more open-minded and try harder to gain additional resources from the organization. Although it is inevitable for them to meet setbacks, their positive psychological resources would exert a protective effect against pressure and stress. They would have more objective or positive evaluations of their workplace and, therefore, would identify more with their organization.

This study also found that income levels were significantly different for residents with different OID. Higher income significantly increased job satisfaction among employees with high OID, and for residents with lower income, the increase in job satisfaction associated with increased OID was not as dramatic as those with higher income. In other words, the effect of OID on job satisfaction is more pronounced for higher-income employees than for lower-income employees. This can be explained through the resource conservation theory. Resource conservation theory suggests that individuals tend to acquire, maintain, and protect the resources they value (Hobfoll, 2001). Wages are one of the valuable resources that organizations give to their employees. Those who get more of this resource tend to be more committed to their organizations and, as a result, are more satisfied with their jobs. On the other hand, employees who earn less may be disappointed with their wages, making their level of identification with the organization have a minor impact on job satisfaction.

### Theoretical Implications

Our findings extend the research on PsyCap and make at least three important contributions to the literature. First, our study expands the research on PsyCap by exploring the relationship between PsyCap and OID. We take PsyCap and OID into consideration simultaneously to explain their influence on job satisfaction, and our results support this hypothesis closely. This means that it is worthwhile to take into account both the concept of explanation and improvement of employees’ job satisfaction.

Second, our research integrated the broaden-and-build theory of positive emotion and resource conservation theory to reveal how PsyCap affects job satisfaction through OID. Meanwhile, we further the research on positive organizational behavior to the resident population, and investigate how low-income levels affect the relationship between OID and job satisfaction in detail.

Third, few studies have examined the mechanisms underlying the effects of OID on job satisfaction among employees with less worked years. By integrating the OID and income level, we contribute to filling this gap by demonstrating the moderating role of income in such a relationship.

### Practical Implications

The results of this study have important practical implications for the managers of residents, as well as policymakers who develop compensation policies. Residents’ identification with the hospitals they are working for is an important prerequisite for improving their job satisfaction, and improving OID is an urgent issue to be addressed by managers who are concerned about the work attitudes of medical staff. First, providing PsyCap training to residents (especially new residents) can help them establish their OID and thus further improve their job satisfaction. The effectiveness of training in PsyCap has been demonstrated in past studies; for example, the daily online self-learning approach was shown to be effective in helping employees improve their PsyCap levels (Da et al., 2020). In addition to training, our research findings highlight the importance of improving how residents are treated.

For compensation developers, there is a need to consider residents’ work stress and ensure that their income matches their workload. Particularly during the global outbreak of COVID-19, doubling workload with lower compensation than other medical staff may have a negative impact on the physical and mental health of residents. Therefore, there is an urgent need to improve resident compensation packages.

### Limitations and Future Directions

First, due to the cross-sectional nature of this study, a causal relationship could not be identified. The second limitation is that this study only utilized self-report measures. Peer reports and objective evaluation indexes should be considered in future research. Third, the job satisfaction was measured using a single item, making it impossible to test the reliability and validity of this variable. Fourth, the generalizability of the construct and related scale of organizational identity in the Chinese cultural context is subject to our sample choice. Due to the training regulations of the residents, most of the sample in this study consisted of young adults who are in the process of career adjustment. They are still changing and developing their organizational identification, which may affect the results of the study. Despite its exploratory nature, this study offers some insight into the relation among PsyCap, OID, and job satisfaction. More longitudinal studies are needed to validate the relationship among them in the future.

## Conclusion

This study highlights the importance of PsyCap with respect to residents’ job satisfaction. OID has been proven to be a partial mediator of this relationship. Moreover, income level moderates the relationship between OID and job satisfaction. The influence of OID on job satisfaction was stronger for residents with a higher level of income. Interventions to improve residents’ job satisfaction should be designed from the perspective of increasing their PsyCap. We advise hospital managers and policymakers to increase the income level of residents, so as to improve residents’ organizational identification and job satisfaction.

## Data Availability Statement

The raw data supporting the conclusions of this article will be made available by the authors, without undue reservation.

## Ethics Statement

The studies involving human participants were reviewed and approved by the Institutional Review Board of Capital Medical University. The patients/participants provided their written informed consent to participate in this study.

## Author Contributions

FZ: investigation, data curation, writing—original draft, and writing—review and editing. YL: conceptualization, formal analysis, methodology, project administration, writing—original draft, and writing—review and editing. TW: writing—review and editing. All authors contributed to the article and approved the submitted version.

## Conflict of Interest

The authors declare that the research was conducted in the absence of any commercial or financial relationships that could be construed as a potential conflict of interest.

## Publisher’s Note

All claims expressed in this article are solely those of the authors and do not necessarily represent those of their affiliated organizations, or those of the publisher, the editors and the reviewers. Any product that may be evaluated in this article, or claim that may be made by its manufacturer, is not guaranteed or endorsed by the publisher.

## References

[B1] Ashforth B. E. Mael F. (1989). Social identity theory and the organization. *Acad. Manag. Rev.* 14:20. 10.2307/258189

[B2] Bandura A. (1997). *Self-Efficacy: The Exercise of Control.* New York, NY: W.H. Freeman.

[B3] Barattucci M. Teresi M. Pietroni D. Iacobucci S. Lo Presti A. Pagliaro S. (2021). Ethical Climate (s), distributed leadership, and work outcomes: the mediating role of organizational identification. *Front. Psychol.* 11:11. 10.3389/fpsyg.2020.564112 33613349 PMC7889511

[B4] Brenner V. C. Carmack C. W. Weinstein M. G. (1971). An empirical test of the motivation-hygiene theory. *J. Account. Res.* 9 359–366.

[B5] Chen K.-Y. Yang C.-M. Lien C.-H. Chiou H.-Y. Lin M.-R. Chang H.-R. (2013). Burnout, job satisfaction, and medical malpractice among physicians. *Int. J. Med. Sci.* 10 1471–1478. 10.7150/ijms.6743 24046520 PMC3775103

[B6] Chen Q. Wen Z. Kong Y. Niu J. Hau K.-T. (2017). Influence of leaders’ psychological capital on their followers: multilevel mediation effect of organizational identification. *Front. Psychol.* 8:1776. 10.3389/fpsyg.2017.01776 29075218 PMC5643467

[B7] Clark A. E. Oswald A. J. (1996). Satisfaction and comparison income. *J. Public. Econ.* 61 359–381. 10.1016/0047-2727(95)01564-7

[B8] Cooper C. L. Rout U. Faragher B. (1989). Mental health, job satisfaction, and job stress among general practitioners. *BMJ* 298 366–370. 10.1136/bmj.298.6670.366 2493939 PMC1835734

[B9] Da S. He Y. Zhang X. (2020). Effectiveness of psychological capital intervention and its influence on work-related attitudes: daily online self-learning method and randomized controlled trial design. *Int. J. Environ. Res. Public Health* 17:8754. 10.3390/ijerph17238754 33255704 PMC7728090

[B10] Dolbier C. L. Webster J. A. McCalister K. T. Mallon M. W. Steinhardt M. A. (2005). Reliability and validity of a single-item measure of job satisfaction. *Am. J. Health Promot.* 19 194–198. 10.4278/0890-1171-19.3.194 15693347

[B11] Edwards J. R. Lambert L. S. (2007). Methods for integrating moderation and mediation: a general analytical framework using moderated path analysis. *Psychol. Methods* 12 1–22. 10.1037/1082-989X.12.1.1 17402809

[B12] Edwards M. R. Peccei R. (2010). Perceived organizational support, organizational identification, and employee outcomes: testing a simultaneous multifoci model. *J. Person. Psychol.* 9 17–26. 10.1027/1866-5888/a000007

[B13] Fletcher D. Sarkar M. (2013). Psychological resilience: a review and critique of definitions. concepts, and theory. *Eur. Psychol.* 18 12–23. 10.1027/1016-9040/a000124

[B14] Fornell C. Larcker D. F. (1981). Evaluating structural equation models with unobservable variables and measurement error. *J. Market. Res.* 18 39–50.

[B15] Fredrickson B. L. (2001). The role of positive emotions in positive psychology: the broaden-and-build theory of positive emotions. *Am. Psychol.* 56 218–226. 10.1037//0003-066x.56.3.218 11315248 PMC3122271

[B16] Gupta M. Shaheen M. Reddy P. K. (2017). Impact of psychological capital on organizational citizenship behavior: mediation by work engagement. *J. Manag. Dev.* 36 973–983. 10.1108/JMD-06-2016-0084

[B17] Hayes A. F. (2013). *Introduction to Mediation, Moderation, and Conditional Process Analysis: A Regression-Based Approach.* New York, NY: The Guilford Press.

[B18] Hobfoll S. E. (2001). The influence of culture, community, and the nested-self in the stress process: advancing conservation of resources theory. *Appl. Psychol. Int. Rev.* 50 337–421.

[B19] IsHak W. W. Lederer S. Mandili C. Nikravesh R. Seligman L. Vasa M. (2009). Burnout during residency training: a literature review. *J. Grad. Med. Educ.* 1 236–242. 10.4300/JGME-D-09-00054.1 21975985 PMC2931238

[B20] Iyeke P. (2020). Job satisfaction as a correlate of empathic behaviour among health care providers towards their patients. *Int. J. Healthc.* 7:29. 10.5430/ijh.v7n1p29

[B21] Karanika-Murray M. Duncan N. Pontes H. M. Griffiths M. D. (2015). Organizational identification, work engagement, and job satisfaction. *J. Manag. Psychol.* 30 1019–1033. 10.1108/JMP-11-2013-0359

[B22] Ke J. Sun J. (2014). Employee’s psychological capital on job satisfaction, organizational commitment and turnover intention. *Res. Econ. Manag.* 01 121–128. 10.13502/j.cnki.issn1000-7636.2014.01.032

[B23] Khalid A. Pan F. Li P. Wang W. Ghaffari A. S. (2020). The impact of occupational stress on job burnout among bank employees in pakistan, with psychological capital as a mediator. *Front. Public Health* 7:410. 10.3389/fpubh.2019.00410 32266193 PMC7105872

[B24] Larson M. Luthans F. (2006). Potential added value of psychological capital in predicting work attitudes. *J. Leaders. Organ. Stud.* 13 75–92. 10.1177/10717919070130020601

[B25] Lee E.-S. Park T.-Y. Koo B. (2015). Identifying organizational identification as a basis for attitudes and behaviors: a meta-analytic review. *Psychol. Bull.* 141 1049–1080. 10.1037/bul0000012 25984729

[B26] Loi R. Chan K. W. Lam L. W. (2014). Leader-member exchange, organizational identification, and job satisfaction: a social identity perspective. *J. Occup. Organ. Psychol.* 87 42–61. 10.1111/joop.12028

[B27] Lu L. Liu L. Sui G. Wang L. (2015). The associations of job stress and organizational identification with job satisfaction among chinese police officers: the mediating role of psychological capital. *Int. J. Environ. Res. Public Health* 12 15088–15099. 10.3390/ijerph121214973 26633436 PMC4690909

[B28] Luthans F. (2002). The need for and meaning of positive organizational behavior. *J. Organ. Behav.* 23 695–706. 10.1002/job.165

[B29] Luthans F. Avey J. B. Clapp-Smith R. Li W. (2008a). More evidence on the value of Chinese workers’ psychological capital: a potentially unlimited competitive resource? *Int. J. Hum. Resour. Manag.* 19 818–827.

[B30] Luthans F. Norman S. M. Avolio B. J. Avey J. B. (2008b). The mediating role of psychological capital in the supportive organizational climate—Employee performance relationship. *J. Organ. Behav.* 29 219–238. 10.1002/job.507

[B31] Luthans F. Youssef C. M. Avolio B. J. (2008c). *Psychological Capital: Developing the Human Competitive Edge (Li Chaoping Translate).* Beijing: China Light Industry Press.

[B32] Luthans F. Avolio B. J. Avey J. B. Norman S. M. (2007). Positive psychological capital: measurement and relationship with performance and satisfaction. *Person. Psychol.* 60 541–572. 10.1111/j.1744-6570.2007.00083.x

[B33] Luthans F. Avolio B. J. Walumbwa F. O. Li W. (2005). The psychological capital of chinese workers: exploring the relationship with performance. *Manag. Organ. Rev.* 1 249–271. 10.1111/j.1740-8784.2005.00011.x

[B34] Luthans F. Youssef-Morgan C. M. (2017). Psychological capital: an evidence-based positive approach. *Annu. Rev. Organ. Psychol. Organ. Behav.* 4 339–366. 10.1146/annurev-orgpsych-032516-113324

[B35] Mael F. Ashforth B. E. (1988). “A reconceptualization of organizational identification,” in *Proceedings of the Midwest Academy of Management Meetings* (Briarcliff Manor, NY: Academy of Management), 127C–129C.

[B36] Mael F. A. Tetrick L. E. (1992). Identifying organizational identification. *Educ. Psychol. Meas.* 52 813–824. 10.1177/0013164492052004002

[B37] Martin L. K. (2021). *Medscape Residents Salary and Debt Report 2021.* Available online at: https://www.medscape.com/slideshow/2021-residents-salary-debt-report-6014074#7

[B38] Podsakoff P. M. MacKenzie S. B. Lee J.-Y. Podsakoff N. P. (2003). Common method biases in behavioral research: a critical review of the literature and recommended remedies. *J. Appl. Psychol.* 88 879–903. 10.1037/0021-9010.88.5.879 14516251

[B39] Pulcrano M. Evans S. R. T. Sosin M. (2016). Quality of life and burnout rates across surgical specialties: a systematic review. *JAMA Surg.* 151 970–978. 10.1001/jamasurg.2016.1647 27410167

[B40] Salvatore D. Numerato D. Fattore G. (2018). Physicians’ professional autonomy and their organizational identification with their hospital. *BMC Health Serv. Res.* 18:775. 10.1186/s12913-018-3582-z 30314481 PMC6186093

[B41] Schneider B. J. Ehsanian R. Schmidt A. Huynh L. Kennedy D. J. Maher D. P. (2021). The effect of patient satisfaction scores on physician job satisfaction and burnout. *Future Sci. OA* 7:FSO657. 10.2144/fsoa-2020-0136 33437508 PMC7787140

[B42] Segars A. H. (1997). Assessing the unidimensionality of measurement: a paradigm and illustration within the context of information systems research. *Omega* 25 107–121.

[B43] Sharma J. P. Bajpai N. (2011). Salary satisfaction as an antecedent of job satisfaction: development of a regression model to determine the linearity between salary satisfaction and job satisfaction in a public and a private organization. *Eur. J. Soc. Sci.* 18 450–461.

[B44] Snyder C. R. Irving L. Anderson J. (1991). “Hope and health: measuring the will and the ways,” in *Handbook of Social and Clinical Psychology*, eds Snyder C. R. Forsyth D. R. (Elmsford, NY: Pergamon), 285–305.

[B45] Spector P. E. (1985). Measurement of human service staff satisfaction: development of the Job Satisfaction Survey. *Am. J. Commun. Psychol.* 13 693–713. 10.1007/BF00929796 4083275

[B46] Stajkovic A. D. Luthans F. (1998). Social cognitive theory and self-efficacy: goin beyond traditional motivational and behavioral approaches. *Organ. Dyn.* 26 62–74. 10.1016/S0090-2616(98)90006-7

[B47] Tian F. Shu Q. Cui Q. Wang L. Liu C. Wu H. (2020). The mediating role of psychological capital in the relationship between occupational stress and fatigue: a cross-sectional study among 1,104 chinese physicians. *Front. Public Health* 8:12. 10.3389/fpubh.2020.00012 32185156 PMC7058796

[B48] Van Dick R. Christ O. Stellmacher J. Wagner U. Ahlswede O. Grubba C. (2004). Should I stay or should I go? Explaining turnover intentions with organizational identification and job satisfaction. *Br. J. Manag.* 15 351–360.

[B49] van Knippenberg D. Sleebos E. (2006). Organizational identification versus organizational commitment: self-definition, social exchange, and job attitudes. *J. Organ. Behav.* 27 571–584. 10.1002/job.359

[B50] Verbrugge L. M. (1982). Work satisfaction and physical health. *J. Commun. Health* 7 262–283. 10.1007/BF01318959 7130446

[B51] Vuong B. Tung D. Tushar H. Quan T. Giao H. (2021). Determinates of factors influencing job satisfaction and organizational loyalty. *Manag. Sci. Lett.* 11 203–212.

[B52] Wang Y. Zheng Y. Zhu Y. (2018). How transformational leadership influences employee voice behavior: the roles of psychological capital and organizational identification. *Soc. Behav. Person.* 46 313–321. 10.2224/sbp.6619

[B53] Wanous J. P. Reichers A. E. Hudy M. J. (1997). Overall job satisfaction: how good are single-item measures? *J. Appl. Psychol.* 82 247–252. 9109282 10.1037/0021-9010.82.2.247

[B54] Williams E. S. Konrad T. R. Scheckler W. E. Pathman D. E. Linzer M. McMurray J. E. (2001). Understanding physicians: intentions to withdraw from practice: the role of job satisfaction, job stress, mental and physical health. *Adv. Health Care Manag.* 2 243–262. 10.1016/S1474-8231(01)02029-8 11233355

[B55] Wu T. Zhang Z. (2017). Do employees with positive psychological have more identification with the organization? The effect of psychological resilience on organizational identity under the perspective of fit. *Finance Trade Res.* 28 101–109.

[B56] Yang W. Chao L. (2016). How psychological contract breach influences organizational identification and organizational citizenship behavior: the mediating role of psychological capital. *Am. J. Ind. Bus. Manag.* 6 922–930. 10.4236/ajibm.2016.68089

[B57] Yu J. Zou F. Sun Y. (2020). Job satisfaction, engagement, and burnout in the population of orthopedic surgeon and neurosurgeon trainees in mainland China. *Neurosurg. Focus* 48;E3. 10.3171/2019.12.FOCUS19830 32114559

